# Dynamics of immune response and drug resistance in malaria infection

**DOI:** 10.1186/1475-2875-5-86

**Published:** 2006-10-11

**Authors:** David Gurarie, F Ellis McKenzie

**Affiliations:** 1 Department of Mathematics, Case Western Reserve University, Cleveland, OH. 44106, USA; 2 Fogarty International Center, Building 16, National Institutes of Health, Bethesda, MD 20892, USA

## Abstract

**Background:**

Malaria parasites that concurrently infect a host compete on the basis of their intrinsic growth rates and by stimulating cross-reactive immune responses that inhibit each others' growth. If the phenotypes also show different drug sensitivities ('sensitive' vs. 'resistant' strains), drug treatment can change their joint dynamics and the long-term outcome of the infection: most obviously, persistent drug pressure can permit the more resistant, but otherwise competitively-inferior, strains to dominate.

**Methods:**

Here a mathematical model is developed to analyse how these and more subtle effects of antimalarial drug use are modulated by immune response, repeated re-inoculation of parasites, drug pharmacokinetic parameters, dose and treatment frequency.

**Results:**

The model quantifies possible effects of single and multiple (periodic) treatment on the outcome of parasite competition. In the absence of further inoculation, the dosage and/or treatment frequency required for complete clearance can be estimated. With persistent superinfection, time-average parasite densities can be derived in terms of the basic immune-regulating parameters, the drug efficacy and treatment regimen.

**Conclusion:**

The functional relations in the model are applicable to a wide range of conditions and transmission environments, allowing predictions to be made on both the individual and the community levels, and, in particular, transitions from drug-sensitive to drug-resistant parasite dominance to be projected on both levels.

## Background

Since the 1920s it has been clear that interactions of immune-system and drug dynamics are critical to the elimination or persistence of drug-resistant malaria parasites in individual patients, but details and dynamic principles remain to be established [1,2]. Later, in the 1980s, it was found that an isolate from a malaria infection is typically a mixture of parasite genotypes, some of which differ in antigenic profile, growth rate and drug response, and show complex population dynamics in mixed cultures [3,4]. Here a mathematical model is developed to investigate the dynamics of parasite phenotypes in a malaria-infected host, with respect to critical interactions between their immune-mediated competition, relative drug sensitivities and persistent superinfection. The results characterize conditions under which a malaria infection dominated by relatively drug-sensitive parasites transitions to one dominated by relatively drug-resistant parasites.

Antimalarial drugs taken prophylactically or early in infection limit the development of immune responses protective against clinical attacks in subsequent infections [5,6]. In endemic regions, antimalarial drug treatment is usually meant to cure a symptomatic malaria infection, thus, usually, a host with incomplete clinical immunity. Hosts are repeatedly re-infected and superinfected with mixtures of parasites. Within an infection, in the absence of drug, the dynamics of the competing parasites are determined in large part by their differences and similarities in stimulating and succumbing to the host's immune responses. The frequency with which drug clears parasites, including drug-resistant parasites, increases with host age, a surrogate for exposure and acquired immunity [7,8]. Stronger, more diverse antibody responses are associated with greater success in antimalarial drug treatment [9,10]. Thus, for instance, all else equal, both a higher prevalence of drug-resistant parasites and a higher frequency of drug treatment would be expected in younger age groups.

Understanding the aggregate effects of immunity can help to guide the introduction of new antimalarial drugs, explain discrepancies between in-vitro and in-vivo tests in drug-resistance surveys [11], and, in general, inform population-based intervention strategies. Indeed, these community patterns summarize very broad effects of immunity across many infections, so their interpretation and application can be greatly improved by a more detailed understanding of immune-drug-parasite interactions within individual infections.

Most studies of intra-host parasite-drug dynamics focus on the clearing effect of drug (the key medical objective) or on optimal strategies for treatment (dosage, frequency). These issues become less relevant under persistent superinfection in endemic areas, however. The focus here is more on longer-term outcomes of treatment (or prophylaxis), and their effects on competition among parasites. For instance, when joined to a proper model of parasite transmission, the crude estimates derived in relating the mean parasitaemia of phenotypes in a 'typical' host to drug dose and frequency should allow community-level analysis of the spread and prevalence of drug -resistant parasites.

The model developed here builds on earlier models of the within-host dynamics of malaria [12,13] to address critical interactions between immune response and drug resistance. In the baseline model, the relative cross-reactivities, immune efficiencies and growth rates of competing parasites determine their dynamics and joint equilibria. Further developments examine the effects of repeated re-inoculations of parasites, then incorporate differences in drug sensitivity between the competing parasites, with the costs of resistance summarized by differences in intrinsic growth rate, to examine how dose, decay rate and frequency of drug administration affect competitive dynamics.

## Methods

### Model of intra-host competition mediated by immunity

The basic model of intra-host competition of multiple parasite phenotypes is based on the models of cross-reactive 'stimulating-clearing' immunity formulated in previous papers [12,13]. Briefly, it describes interactions in which parasite phenotypes stimulate the production of non-specific and specific immune effectors, which clear (kill) them at rates that depend on the strength, specificity and quantity of effector.

Mathematically the model is a coupled system of differential equations for parasite densities *x*, *y*, and immune-effector densities: non-specific *I *and specific *J*,*K *for *x*,*y*-species respectively (see [12,13]),

x˙=[aX−f(xxp+yyp)−cnI−cX(J+ηK)]x+SX;y˙=[aY−f(xxp+yyp)−cnI−cY(ξJ+K)]y+SY;     (1)I˙=σn(x+y)−μnI;J˙=σXx−μsJ;K˙=σYy−μsK;
 MathType@MTEF@5@5@+=feaafiart1ev1aaatCvAUfKttLearuWrP9MDH5MBPbIqV92AaeXatLxBI9gBaebbnrfifHhDYfgasaacH8akY=wiFfYdH8Gipec8Eeeu0xXdbba9frFj0=OqFfea0dXdd9vqai=hGuQ8kuc9pgc9s8qqaq=dirpe0xb9q8qiLsFr0=vr0=vr0dc8meaabaqaciaacaGaaeqabaqabeGadaaakqaabeqaaiqbdIha4zaacaGaeyypa0ZaamWaaeaacqWGHbqydaWgaaWcbaGaemiwaGfabeaakiabgkHiTiabdAgaMnaabmaabaWaaSaaaeaacqWG4baEaeaacqWG4baEdaWgaaWcbaGaemiCaahabeaaaaGccqGHRaWkdaWcaaqaaiabdMha5bqaaiabdMha5naaBaaaleaacqWGWbaCaeqaaaaaaOGaayjkaiaawMcaaiabgkHiTiabdogaJnaaBaaaleaacqWGUbGBaeqaaOGaemysaKKaeyOeI0Iaem4yam2aaSbaaSqaaiabdIfaybqabaGcdaqadaqaaiabdQeakjabgUcaRGGaciab=D7aOHqaciab+TealbGaayjkaiaawMcaaaGaay5waiaaw2faaiabdIha4jabgUcaRiabdofatnaaBaaaleaacqWGybawaeqaaOGaei4oaSdabaGafmyEaKNbaiaacqGH9aqpdaWadaqaaiabdggaHnaaBaaaleaacqWGzbqwaeqaaOGaeyOeI0IaemOzay2aaeWaaeaadaWcaaqaaiabdIha4bqaaiabdIha4naaBaaaleaacqWGWbaCaeqaaaaakiabgUcaRmaalaaabaGaemyEaKhabaGaemyEaK3aaSbaaSqaaiabdchaWbqabaaaaaGccaGLOaGaayzkaaGaeyOeI0Iaem4yam2aaSbaaSqaaiabd6gaUbqabaGccqWGjbqscqGHsislcqWGJbWydaWgaaWcbaGaemywaKfabeaakmaabmaabaGae8NVdGNaemOsaOKaey4kaSIaem4saSeacaGLOaGaayzkaaaacaGLBbGaayzxaaGaemyEaKNaey4kaSIaem4uam1aaSbaaSqaaiabdMfazbqabaGccqGG7aWocaWLjaGaaCzcamaabmaabaGaeGymaedacaGLOaGaayzkaaaabaGafmysaKKbaiaacqGH9aqpcqWFdpWCdaWgaaWcbaGaemOBa4gabeaakmaabmaabaGaemiEaGNaey4kaSIaemyEaKhacaGLOaGaayzkaaGaeyOeI0Iae8hVd02aaSbaaSqaaiabd6gaUbqabaGccqWGjbqscqGG7aWocuWGkbGsgaGaaiabg2da9iab=n8aZnaaBaaaleaacqWGybawaeqaaOGaemiEaGNaeyOeI0Iae8hVd02aaSbaaSqaaiabdohaZbqabaGccqWGkbGscqGG7aWocuWGlbWsgaGaaiabg2da9iab=n8aZnaaBaaaleaacqWGzbqwaeqaaOGaemyEaKNaeyOeI0Iae8hVd02aaSbaaSqaaiabdohaZbqabaGccqWGlbWscqGG7aWoaaaa@B052@

In the follow up analysis of drug treatment *x *will represent a resistant strain, while *y *-a sensitive one. This is similar to the basic model of [12] but augmented with two additional features:

(i) a 'fever regulator' *f*(*z*) = *F*_0_*φ*_*q*_(*z*), described by a sigmoid function *φ*_*q*_(*z*) = *z*^*q*^/(1 + *z*^*q*^), switched 'on' and 'off by the combined parasite density (over their pyrogenic thresholds *x*_*p*_, *y*_*p*_), with maximal pyrogenic removal rate *F*_0_.

(ii) super-infection (inoculation) sources *S*_*X*_;*S*_*Y*_, which can be either stationary (convenient for qualitative analysis and numeric simulation), or stochastic with a prescribed 'spacing distribution' between inoculations.

The fever plays a limited role in the dynamics of (1). At the initial stage of infection it serves to arrest the parasite growth about the pyrogenic threshold, while immune effectors build up, but then becomes negligible below that level. Therefore it is omitted in the analyses of subfebrile equilibria, but is included in the dynamic simulations.

The immune-stimulation terms (in the *I*,*J*,*K *– equations) are taken here as simple linear functions, so that the effectors are stimulated in proportion to parasite densities. Other parameters in system (1) are similar to [12,13]:

(i) Parasite growth rates: *a*_*X*_;*a*_*Y*_

(ii) Stimulation coefficients, for nonspecific and specific effectors: *σ*_*n*_;*σ*_*X*_;*σ*_*Y*_

(iii) Clearing coefficients, for nonspecific and specific effectors: *c*_*n*_;*c*_*X*_;*c*_*Y*_

(iv) Decay/deactivation rates, for nonspecific and specific effectors: *μ*_*n*_;*μ*_*s*_

(v) Cross-reactivities, in terms of the relative clearing rates of the x-effector on y and the y-effector on x: 0 ≤ *ξ*, *η *< 1

System (1) can be rescaled to a nondimensional form which yields the essential (non-dimensional) parameters. The most important of those are *cross-reactivities *and the *immune efficiencies *(non-specific *e*_*n *_and specific *e*_*x*_, *e*_*y*_) introduced in [13]. The immune efficiencies combine the appropriate 'stimulation' and 'clearing' coefficients against the product of 'immune decay' and 'parasite growth' into a single nondimensional parameter.

System (1) can be considered a 'multi-prey, multi-predator' model of parasites and immune effectors, or alternatively as 'immune-mediated' competition between parasites *x *and *y*. The outcome of such competition depends on phenotypic traits with respect to the host immune response, as expressed through the essential, non-dimensional parameters above (cross-reactivities and immune efficiencies), as well as *relative parasite growth rate α *= *a*_*Y*_/*a*_*X*_. Note, for instance, that different parasite species may differ in replication rates (*a*_*X *_≠ *a*_*Y*_) and show low cross-reactivity, while different "strains" of a species (e.g. drug-sensitive or drug-resistant/tolerant) may be more strongly cross-reactive

### Basic pharmaco-dynamics of drug treatment

The next development is a simple pharmaco-dynamic model for a single parasite phenotype. The degree to which drug treatment reduces the growth rate of a given parasite depends on both the pharmacokinetic characteristics of the drug and the sensitivity of the parasite to the drug. Key assumptions are that the phenotypic cost of drug resistance is a lower intrinsic growth rate in the absence of drug, and that the drug-induced reduction can be approximated from the basic drug parameters and the frequency of (periodic) treatment. The effect of 'heavy treatment' may be to remove the more sensitive strain completely, or, for repeated treatment, with superinfection, to tip the effective relative growth rate *α *in favour of *x *(below its critical levels *α*_1,2_, explained in the following sections), releasing the more resistant strain from competitive constraints.

The pharmacodynamic model adopts the basic premises of [14]:

(i) after intake, drug concentration decays from its initial dose *d*_0 _at an exponential rate *d*(*t*) = *d*_0_*e*^-*βt *^(for simplicity, blood and tissue decay are not distinguished);

(ii) the drug removes parasites through a 'clearing function' *Bφ*_*p*_(*d*/*d*_*S*_), where *B *denotes the maximal clearing rate, *d*_*S *_the parasite 'sensitivity threshold' (often denoted *d*_50_, as it clears parasite at 50% maximal rate), and *φ*_*p*_(*z*) – a sigmoid function *φ *= *φ*_*p*_(*z*) = *z*^*p*^/(1 + *z*^*p*^) with a suitable Hill exponent *p*.

A large *p *in *φ *means that the clearing rate remains relatively steady ≈ *B *over a wide range of drug concentrations, provided that *d *stays above *d*_50_. For the numeric simulations below, the exponent *p *= 3, close to its estimated value for mefloquine [15].

The resulting dynamic treatment model [14] with constant parasite growth and a source (inoculation) *S*, takes the form

x˙
 MathType@MTEF@5@5@+=feaafiart1ev1aaatCvAUfKttLearuWrP9MDH5MBPbIqV92AaeXatLxBI9gBaebbnrfifHhDYfgasaacH8akY=wiFfYdH8Gipec8Eeeu0xXdbba9frFj0=OqFfea0dXdd9vqai=hGuQ8kuc9pgc9s8qqaq=dirpe0xb9q8qiLsFr0=vr0=vr0dc8meaabaqaciaacaGaaeqabaqabeGadaaakeaacuWG4baEgaGaaaaa@2E2E@ = *a*[1 - *bφ*(*D*(*t*))]*x *+ *S*(*t*)     (2)

where *D*(*t*) = *d*(*t*)/*d*_*S *_is the dimensionless drug concentration, and *b *= *B*/*a *the relative parasite clearing rate. Two special cases are:

(i) *D*(*t*) = D0e−β(t−t0)
 MathType@MTEF@5@5@+=feaafiart1ev1aaatCvAUfKttLearuWrP9MDH5MBPbIqV92AaeXatLxBI9gBaebbnrfifHhDYfgasaacH8akY=wiFfYdH8Gipec8Eeeu0xXdbba9frFj0=OqFfea0dXdd9vqai=hGuQ8kuc9pgc9s8qqaq=dirpe0xb9q8qiLsFr0=vr0=vr0dc8meaabaqaciaacaGaaeqabaqabeGadaaakeaacqWGebardaWgaaWcbaGaeGimaadabeaakiabdwgaLnaaCaaaleqabaGaeyOeI0ccciGae8NSdi2aaeWaaeaacqWG0baDcqGHsislcqWG0baDdaWgaaadbaGaeGimaadabeaaaSGaayjkaiaawMcaaaaaaaa@3974@ – for a single dose administered at *t*_0_, written in terms of a dimensionless initial dose *D*_0 _= *d*_0_/*d*_*S *_≫ 1;

(ii) a periodic function (equation 5; presented below) for multiple applications with period *T*.

Equation (2) contains the essential *pharmaco-kinetics *of drug (the relative parasite clearing rate *b *and 'clearing pattern' *φ*), as well as *its pharmaco-dynamics *(initial dose and the frequency of treatment encoded in *D*(*t*)).

Drug treatment will enter system (1) or its rescaled version (3) as additional attrition terms: *b*_*X*/*Y*_*φ*(*D*_*X*/*Y*_(*t*)) in the *x*, *y *equations. Two phenotypes are assumed to have different drug-sensitivities, with *x *the more tolerant/resistant and *y *the more sensitive strain. Hence the different clearing coefficients, *b*_*X *_<*b*_*Y *_(to indicate higher maximal removal rate of *y*), and different drug-thresholds, expressed through their relative initial dosages, *D*_*X *_<*D*_*Y *_(*y *being cleared at lower concentrations of drug, compared to *x*). Thus strain y has a higher natural growth rate, *α *= *a*_*Y*_/*a*_*x *_> 1, and is competitively superior in the absence of drug, but, as shown below, drug treatment can offset this advantage.

## Results

### Analysis of stationary equilibria

The analysis at this stage is meant to outline the long-term effect of parasite-immune interactions. In particular, it attempts to predict the resulting parasite densities (long term 'outcome of competition') through the basic host parameters (growth rates, immune efficiencies, cross-reactivities), and does not yet involve drug treatment.

The rescaled system (1) with drug treatment represented by time-dependent concentration *D *= *D*(*t*) is given by a coupled differential system

x˙=aX[1−f(x+yyp)−bXφ(D)−enI−eX(J+ηK)]x+S1;y˙=aX[α−f(x+yyp)−bYφ(D)−enI−eY(ξJ+K)]y+S2;     (3)I˙=μn[(x+y)−I];J˙=μs(x−J);K˙=μs(y−K);
 MathType@MTEF@5@5@+=feaafiart1ev1aaatCvAUfKttLearuWrP9MDH5MBPbIqV92AaeXatLxBI9gBaebbnrfifHhDYfgasaacH8akY=wiFfYdH8Gipec8Eeeu0xXdbba9frFj0=OqFfea0dXdd9vqai=hGuQ8kuc9pgc9s8qqaq=dirpe0xb9q8qiLsFr0=vr0=vr0dc8meaabaqaciaacaGaaeqabaqabeGadaaakqaabeqaaiqbdIha4zaacaGaeyypa0Jaemyyae2aaSbaaSqaaiabdIfaybqabaGcdaWadaqaaiabigdaXiabgkHiTiabdAgaMnaabmaabaGaemiEaGNaey4kaSYaaSaaaeaacqWG5bqEaeaacqWG5bqEdaWgaaWcbaGaemiCaahabeaaaaaakiaawIcacaGLPaaacqGHsislcqWGIbGydaWgaaWcbaGaemiwaGfabeaaiiGakiab=z8aMnaabmaabaGaemiraqeacaGLOaGaayzkaaGaeyOeI0Iaemyzau2aaSbaaSqaaiabd6gaUbqabaGccqWGjbqscqGHsislcqWGLbqzdaWgaaWcbaGaemiwaGfabeaakmaabmaabaGaemOsaOKaey4kaSIae83TdGMaem4saSeacaGLOaGaayzkaaaacaGLBbGaayzxaaGaemiEaGNaey4kaSIaem4uam1aaSbaaSqaaiabigdaXaqabaGccqGG7aWoaeaacuWG5bqEgaGaaiabg2da9iabdggaHnaaBaaaleaacqWGybawaeqaaOWaamWaaeaacqWFXoqycqGHsislcqWGMbGzdaqadaqaaiabdIha4jabgUcaRmaalaaabaGaemyEaKhabaGaemyEaK3aaSbaaSqaaiabdchaWbqabaaaaaGccaGLOaGaayzkaaGaeyOeI0IaemOyai2aaSbaaSqaaiabdMfazbqabaGccqWFgpGzdaqadaqaaiabdseaebGaayjkaiaawMcaaiabgkHiTiabdwgaLnaaBaaaleaacqWGUbGBaeqaaOGaemysaKKaeyOeI0Iaemyzau2aaSbaaSqaaiabdMfazbqabaGcdaqadaqaaiab=57a4jabdQeakjabgUcaRiabdUealbGaayjkaiaawMcaaaGaay5waiaaw2faaiabdMha5jabgUcaRiabdofatnaaBaaaleaacqaIYaGmaeqaaOGaei4oaSJaaCzcaiaaxMaadaqadaqaaiabiodaZaGaayjkaiaawMcaaaqaaiqbdMeajzaacaGaeyypa0Jae8hVd02aaSbaaSqaaiabd6gaUbqabaGcdaWadaqaamaabmaabaGaemiEaGNaey4kaSIaemyEaKhacaGLOaGaayzkaaGaeyOeI0IaemysaKeacaGLBbGaayzxaaGaei4oaSJafmOsaOKbaiaacqGH9aqpcqWF8oqBdaWgaaWcbaGaem4CamhabeaakmaabmaabaGaemiEaGNaeyOeI0IaemOsaOeacaGLOaGaayzkaaGaei4oaSJafm4saSKbaiaacqGH9aqpcqWF8oqBdaWgaaWcbaGaem4CamhabeaakmaabmaabaGaemyEaKNaeyOeI0Iaem4saSeacaGLOaGaayzkaaGaei4oaSdaaaa@B745@

It depends on several dimensionless parameters: *fever efficiency *f0=F0aX
 MathType@MTEF@5@5@+=feaafiart1ev1aaatCvAUfKttLearuWrP9MDH5MBPbIqV92AaeXatLxBI9gBaebbnrfifHhDYfgasaacH8akY=wiFfYdH8Gipec8Eeeu0xXdbba9frFj0=OqFfea0dXdd9vqai=hGuQ8kuc9pgc9s8qqaq=dirpe0xb9q8qiLsFr0=vr0=vr0dc8meaabaqaciaacaGaaeqabaqabeGadaaakeaacqWGMbGzdaWgaaWcbaGaeGimaadabeaakiabg2da9maalaaabaGaemOray0aaSbaaSqaaiabicdaWaqabaaakeaacqWGHbqydaWgaaWcbaGaemiwaGfabeaaaaaaaa@3524@, *cross-reactivities *0 ≤ *ξ*, *η *< 1, and immune efficiencies: en=cnσnaXμnxp
 MathType@MTEF@5@5@+=feaafiart1ev1aaatCvAUfKttLearuWrP9MDH5MBPbIqV92AaeXatLxBI9gBaebbnrfifHhDYfgasaacH8akY=wiFfYdH8Gipec8Eeeu0xXdbba9frFj0=OqFfea0dXdd9vqai=hGuQ8kuc9pgc9s8qqaq=dirpe0xb9q8qiLsFr0=vr0=vr0dc8meaabaqaciaacaGaaeqabaqabeGadaaakeaacqWGLbqzdaWgaaWcbaGaemOBa4gabeaakiabg2da9maalaaabaGaem4yam2aaSbaaSqaaiabd6gaUbqabaacciGccqWFdpWCdaWgaaWcbaGaemOBa4gabeaaaOqaaiabdggaHnaaBaaaleaacqWGybawaeqaaOGae8hVd02aaSbaaSqaaiabd6gaUbqabaaaaOGaemiEaG3aaSbaaSqaaiabdchaWbqabaaaaa@4013@, eX=cXσXaXμsxp,eY=cYσYaXμsxp
 MathType@MTEF@5@5@+=feaafiart1ev1aaatCvAUfKttLearuWrP9MDH5MBPbIqV92AaeXatLxBI9gBaebbnrfifHhDYfgasaacH8akY=wiFfYdH8Gipec8Eeeu0xXdbba9frFj0=OqFfea0dXdd9vqai=hGuQ8kuc9pgc9s8qqaq=dirpe0xb9q8qiLsFr0=vr0=vr0dc8meaabaqaciaacaGaaeqabaqabeGadaaakeaacqWGLbqzdaWgaaWcbaGaemiwaGfabeaakiabg2da9maalaaabaGaem4yam2aaSbaaSqaaiabdIfaybqabaacciGccqWFdpWCdaWgaaWcbaGaemiwaGfabeaaaOqaaiabdggaHnaaBaaaleaacqWGybawaeqaaOGae8hVd02aaSbaaSqaaiabdohaZbqabaaaaOGaemiEaG3aaSbaaSqaaiabdchaWbqabaGccqGGSaalcqWGLbqzdaWgaaWcbaGaemywaKfabeaakiabg2da9maalaaabaGaem4yam2aaSbaaSqaaiabdMfazbqabaGccqWFdpWCdaWgaaWcbaGaemywaKfabeaaaOqaaiabdggaHnaaBaaaleaacqWGybawaeqaaOGae8hVd02aaSbaaSqaaiabdohaZbqabaaaaOGaemiEaG3aaSbaaSqaaiabdchaWbqabaaaaa@536A@. To facilitate analysis we drop the fever term, assuming 'subfebrile equilibria' x*, y* ≪ 1 that result from (relatively) high immune efficiencies *e*_*n*_;*e*_*X*,*Y *_≫ 1. The equilibrium equations for (3) without treatment or fever, are then reduced to the classical Volterra-Lotka system

F(x,y)=[1−(eX+en)x−(ηeX+en)y]x+SX=0G(x,y)=[α−(ξeY+en)x−(eY+en)y]y+SY=0     (4)
 MathType@MTEF@5@5@+=feaafiart1ev1aaatCvAUfKttLearuWrP9MDH5MBPbIqV92AaeXatLxBI9gBaebbnrfifHhDYfgasaacH8akY=wiFfYdH8Gipec8Eeeu0xXdbba9frFj0=OqFfea0dXdd9vqai=hGuQ8kuc9pgc9s8qqaq=dirpe0xb9q8qiLsFr0=vr0=vr0dc8meaabaqaciaacaGaaeqabaqabeGadaaakeaafaqabeGabaaabaGaemOray0aaeWaaeaacqWG4baEcqGGSaalcqWG5bqEaiaawIcacaGLPaaacqGH9aqpdaWadaqaaiabigdaXiabgkHiTmaabmaabaGaemyzau2aaSbaaSqaaiabdIfaybqabaGccqGHRaWkcqWGLbqzdaWgaaWcbaGaemOBa4gabeaaaOGaayjkaiaawMcaaiabdIha4jabgkHiTmaabmaabaacciGae83TdGMaemyzau2aaSbaaSqaaiabdIfaybqabaGccqGHRaWkcqWGLbqzdaWgaaWcbaGaemOBa4gabeaaaOGaayjkaiaawMcaaiabdMha5bGaay5waiaaw2faaiabdIha4jabgUcaRiabdofatnaaBaaaleaacqWGybawaeqaaOGaeyypa0JaeGimaadabaGaem4raC0aaeWaaeaacqWG4baEcqGGSaalcqWG5bqEaiaawIcacaGLPaaacqGH9aqpdaWadaqaaiab=f7aHjabgkHiTmaabmaabaGae8NVdGNaemyzau2aaSbaaSqaaiabdMfazbqabaGccqGHRaWkcqWGLbqzdaWgaaWcbaGaemOBa4gabeaaaOGaayjkaiaawMcaaiabdIha4jabgkHiTmaabmaabaGaemyzau2aaSbaaSqaaiabdMfazbqabaGccqGHRaWkcqWGLbqzdaWgaaWcbaGaemOBa4gabeaaaOGaayjkaiaawMcaaiabdMha5bGaay5waiaaw2faaiabdMha5jabgUcaRiabdofatnaaBaaaleaacqWGzbqwaeqaaOGaeyypa0JaeGimaadaaiaaxMaacaWLjaWaaeWaaeaacqaI0aanaiaawIcacaGLPaaaaaa@8137@

**Figure 1:**
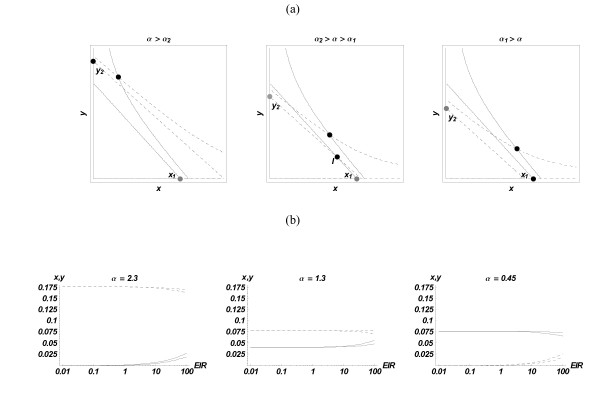
**Phase-plane of dynamical system (4)**. (a) Phase-plane views of 3 regions of system (4) in terms of relative growth rate *α *: *y*-domination (equilibrium *y*_2_, left); coexistence (equilibrium I, middle) and x-domination (equilibrium *x*_1_, right). Stable equilibria are marked in black. In all 3 cases, adding sources (inoculations) produces a stable coexistence equilibrium (intersection of asymptotic hyperbolae). The two solid lines are *x*-nullclines *F*(*x*, *y*) = 0, the two dashed lines are *y*-nullclines *G*(*x*, *y*) = 0. **(b) **Equilibria (4) for stationary sources *S*_*X*,*Y *_(*x*(*ε*) – solid, *y*(*ε*) – dashed), with the parameters of Table 1, in 3 different ranges of *α*, as functions of EIR, 0 <*ε *< 100. Two sets of curves on each plot correspond to different partitions of EIR among strains: (i) *p*_*X *_= *p*_*Y *_= .5 (outer curves), (ii) *p*_*X *_= .9; *p*_*Y *_= .1 (inner '*x*-biased' curves). The two solid lines are x-strains, and the dashed lines are y-strains.

In the absence of sources (*S*_*X*,*Y *_= 0), it has three types of equilibria (Figure 1). Two of them, '*x*-domination' (*x*_1_, 0) or '*y*-domination' (0, *y*_2_), are given by coordinate intercepts of the 'linear factors' in the *F*-equation: *x*_1 _= 1/(*e*_*X *_+ *e*_*n*_); *y*_1 _= 1/(*ηe*_*X *_+ *e*_*n*_), and the *G*-equation *x*_2 _= *α*/(*ξe*_*Y *_+ *e*_*n*_); *y*_2 _= *α*/(*e*_*Y *_+ *e*_*n*_). The third possibility is a state of 'coexistence' (x*, y*). The stability and qualitative behaviour of equilibria depend on the relative position of two null-clines (4). In our case, the equations *y*_1 _= *y*_2 _and *x*_1 _= *x*_2 _give two critical (bifurcation) values of the relative growth-rate (fitness parameter) *α *= *a*_*Y*_/*a*_*X*_, namely

α1=eY+enηeX+en>α2=ξeY+eneX+en     (5)
 MathType@MTEF@5@5@+=feaafiart1ev1aaatCvAUfKttLearuWrP9MDH5MBPbIqV92AaeXatLxBI9gBaebbnrfifHhDYfgasaacH8akY=wiFfYdH8Gipec8Eeeu0xXdbba9frFj0=OqFfea0dXdd9vqai=hGuQ8kuc9pgc9s8qqaq=dirpe0xb9q8qiLsFr0=vr0=vr0dc8meaabaqaciaacaGaaeqabaqabeGadaaakeaaiiGacqWFXoqydaWgaaWcbaGaeGymaedabeaakiabg2da9maalaaabaGaemyzau2aaSbaaSqaaiabdMfazbqabaGccqGHRaWkcqWGLbqzdaWgaaWcbaGaemOBa4gabeaaaOqaaiab=D7aOjabdwgaLnaaBaaaleaacqWGybawaeqaaOGaey4kaSIaemyzau2aaSbaaSqaaiabd6gaUbqabaaaaOGaeyOpa4Jae8xSde2aaSbaaSqaaGGaaiab+jdaYaqabaGccqGH9aqpdaWcaaqaaiab=57a4jabdwgaLnaaBaaaleaacqWGzbqwaeqaaOGaey4kaSIaemyzau2aaSbaaSqaaiabd6gaUbqabaaakeaacqWGLbqzdaWgaaWcbaGaemiwaGfabeaakiabgUcaRiabdwgaLnaaBaaaleaacqWGUbGBaeqaaaaakiaaxMaacaWLjaWaaeWaaeaacqaI1aqnaiaawIcacaGLPaaaaaa@56E4@

The three fitness regions include:

α>α1-'y-domination';α1>α>α2-coexistence;     (6)α2>α-'x-domination'
 MathType@MTEF@5@5@+=feaafiart1ev1aaatCvAUfKttLearuWrP9MDH5MBPbIqV92AaeXatLxBI9gBaebbnrfifHhDYfgasaacH8akY=wiFfYdH8Gipec8Eeeu0xXdbba9frFj0=OqFfea0dXdd9vqai=hGuQ8kuc9pgc9s8qqaq=dirpe0xb9q8qiLsFr0=vr0=vr0dc8meaabaqaciaacaGaaeqabaqabeGadaaakqaabeqaaGGaciab=f7aHjabg6da+iab=f7aHnaaBaaaleaacqaIXaqmaeqaaOGaeeyla0Iaei4jaCIaeeyEaKNaeeyla0IaeeizaqMaee4Ba8MaeeyBa0MaeeyAaKMaeeOBa4MaeeyyaeMaeeiDaqNaeeyAaKMaee4Ba8MaeeOBa4Maei4jaCIaee4oaSdabaGae8xSde2aaSbaaSqaaiabbgdaXaqabaGccqGH+aGpcqWFXoqycqWF+aGpcqWFXoqydaWgaaWcbaaccaGae4Nmaidabeaakiabb2caTiabbogaJjabb+gaVjabbwgaLjabbIha4jabbMgaPjabbohaZjabbsha0jabbwgaLjabb6gaUjabbogaJjabbwgaLjabbUda7iaaxMaacaWLjaWaaeWaaeaacqqG2aGnaiaawIcacaGLPaaaaeaacqWFXoqydaWgaaWcbaGae4Nmaidabeaakiabg6da+iab=f7aHjabb2caTiabbEcaNiabbIha4jabb2caTiabbsgaKjabb+gaVjabb2gaTjabbMgaPjabb6gaUjabbggaHjabbsha0jabbMgaPjabb+gaVjabb6gaUjabbEcaNaaaaa@7B54@

Here 'domination' in the absence of inoculation sources, means complete removal of the competitor. The familiar phase-plots of such competition are illustrated in Figure 1.

The presence of stationary sources of infection *S*_*X*_; *S*_*Y *_> 0 (no matter how small) will shift all three types of equilibria to the upper-right quadrant into a stable 'coexistence state', defined by two asymptotic hyperbolae (solid-x and dashed-y, Figure 1). Now 'domination' does not mean complete removal, but a 'dominant density', e.g. y*/x* ≫ 1. The use of *α *as the 'control parameter' is relevant for qualitative analysis of drug intervention (below). Indeed, given two parasites with different drug-sensitivities, drug treatment effectively lowers their growth rates, and thus tips the outcome of competition in favour of the resistant strain.

This qualitative analysis makes two simplifying assumptions – a reduced 2D model in place of the full 5D (3), and the omission of fever. The latter has minor consequences for high immune efficiencies. The dimensional reduction (5D to 2D) maintains the equilibrium values x*, y*, but changes their stability types somewhat, from '2D stable nodes' (real negative eigenvalues of the Jacobian matrix), to '5D spiral sinks' (complex negative eigenvalues), (see [13] for details).

### Dynamic relaxation

Next, the dynamics of this baseline model are examined by numerically computing solutions of rescaled system (3) with and without inoculation sources, using the parameter values described in Table 1. Some parameter values are based on earlier work, e.g. the relative pyrogenic clearing rate [18] and immune loss rates [12]. The essential 'uncertain parameters' are the immune efficiencies and cross-reactivities. Because the immune efficiencies control equilibria as ≈ 1/(*e*_*n *_+ *e*_*X*,*Y*_) we chose {*e*_*n*_,*e*_*X*,*Y*_} sufficiently large to keep equilibria below pyrogenic levels, as expected for a typical 'asymptomatic parasitaemia'. The exact choice of *e *– values makes little qualitative difference, provided equilibria stay below the 'pyrogenic level' *x*_*p*_, but could affect the pharmacokinetic parameters (doses) for parasite clearing, as explained in the next section.

**Table 1:** Basic parameters for dynamic model (3)

Fever: Hill exponents, clearing efficiency and pyrogenic parasitaemia levels	Immune efficiencies	Cross-reactivities	Immune decay rate/half-life
*q *= l2; *f*_0 _= *F*_0_/*a*_*X *_= 1.2 *x*_*p *_= *y*_*p *_= 1	*e*_*n *_= 3 *e*_*X *_= *e*_*Y *_= 10	*ξ *= .4 *η *= .3	*μ*_*n *_= .45(≈ 1.5 days) *μ*_*s *_= .014(≈ 50 days)

The role of cross-reactivities in the equilibrium analysis above is to narrow (*ξ*, *η *≈ 0) or widen (*ξ*, *η *≫ 0) the *α *- range of coexistence. Intermediate values are chosen to indicate a relative genetic (antigenic) proximity of the competing strains.

Under this choice of parameters the predicted thresholds for the relative growth rate *α *(5)-(6) become *α*_1 _= 2.17; *α*_2 _= .54. Also introduced is a lower cut-off for parasite densities, *x*_*c *_= 10^-11 ^(in dimensionless units), assuming a pyrogenic threshold *x*_0 _= 10^4^/*μl*, corresponding to a density of 10^-7^/*μl *(i.e. fewer than one parasite for the entire blood volume of an adult). If either of the densities *x*,*y *falls below *x*_*c *_it is set to zero – i.e. complete clearing.

The inoculation sources are considered either stationary, of strength EIR × "injected density *s*_0_", or 'random,' with exponentially distributed waiting times and mean spacing EIR ('entomological inoculation rate,' i.e. the frequency of infectious bites by mosquitoes). The 'injected density' is estimated in terms of the parasites (primary merozoites) released from the liver, assuming a mean of 10 parasites (sporozoites) per mosquito inoculum, with each sporozoite developing into 30,000 primary merozoites, thus a total 300,000 per 4.5 liters, or 0.67/ml. This gives a dimensionless value, *s*_0 _= 6.7·10^-5^. The infection source is distributed among the two phenotypes in different proportions meant to reflect parasite prevalences in the community: *p*_*X *_+ *p*_*Y *_= 1. Dynamic simulations with stochastic *S *use relatively low EIR *ε *= .3/day, due to computational constraints, but the use of equivalent stationary (mean) sources allows sampling of a broad range of EIR. Figure 1(b) demonstrates the effect of stationary EIR on x,y-equilibria. It remains marginal for a wide range of EIR, but high values *O*(10 – 100) can bring about a significant shift.

**Figure 2:**
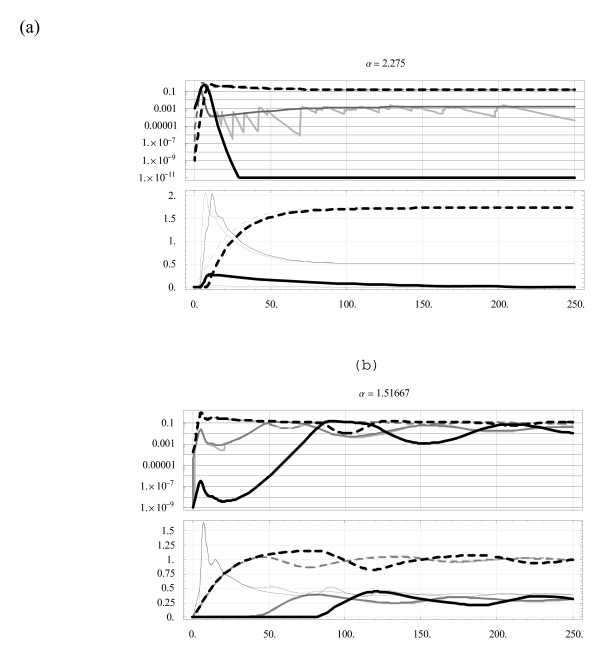
**Dynamic histories**. Three long-term dynamic histories for 2 cases of Figure 1 over the time range 0 <*t *< 250 days (horizontal axis). The top plot in each panel shows dimensionless densities of two strains (*x*(*t*) – solid, *y*(*t*) – dashed), relative to pyrogenic levels *x*_*p *_= *y*_*p *_= 1; the bottom plot shows their immune effectors, *I*(*t*) – thin, *J*(*t*) – solid thick, *K*(*t*) – dashed, weighted by their respective efficiencies *e*_*n*_;*e*_*X*_;*e*_*Y*_. For each panel, the shading (black, heavy gray, light gray) corresponds to the case of 'no-source', 'stationary source', 'stochastic source'. Note that '*y*-curves' (dashed) are essentially the same in all 3 cases, leaving *y *unaffected, while *x *is strongly affected by the 'stationary' and 'random' sources.

Figure 2 shows computed solutions of system (3) with the parameters of Table 1 in two cases: (a) '*y*-dominant' *α *> *α*_1_; (b) *x*,*y *coexistence. Solid curves on each of the upper panels show *x*, and dashed curves *y*; the same marking is used for their specific (SS) effectors on the lower panels, while the non-specific (NS) is marked by thin curves. In each case three solutions are compared, marked in shades of gray: black corresponds to the unperturbed system (i.e. no inoculation), dark gray has steady sources *S*_*X *_= *p*_*X*_*εs*_0_; *S*_*Y *_= *p*_*Y*_*εs*_0_, and light gray has random sources of the same proportions. In case (a) inoculation has a marginal effect on the 'dominant' species, as its equilibrium level *O*(10^-1^) stays far above the source level *O*(10^-4^), but the 'losing side' gains in strength at a level comparable to the effective stationary source (Figure 2(a): 2 gray '*x*-curves'). Indeed, the complete clearance of *x *by day 30 in plot (a) (black 'x-curve') is changed into the 'quasi-equilibrated' value x* ≈ 10^-2^y*. Note also that random inoculation paths closely follow the 'mean inoculation' curves. This observation is used to replace (the computationally extensive) 'stochastic source' with its stationary mean.

For coexistence, in Figure 2(b) the equilibrium values show little change, but the amplitude and phases of 'damped oscillations' are shifted during the initial stage. Overall the dynamic simulations confirm the earlier conclusion that inoculation (stationary or random) will change the outcome of competition to a coexistence pattern, though the two equilibria may differ by orders of magnitude.

### Single drug treatment

The simple growth-treatment model (2) for a single parasite without immune response allows exact analytic solution, expressed through the multiplier function  X(t)=a[t−bβpln⁡(1+D0p1+D0pe−pβt)]
 MathType@MTEF@5@5@+=feaafiart1ev1aaatCvAUfKttLearuWrP9MDH5MBPbIqV92AaeXatLxBI9gBaebbnrfifHhDYfgasaacH8akY=wiFfYdH8Gipec8Eeeu0xXdbba9frFj0=OqFfea0dXdd9vqai=hGuQ8kuc9pgc9s8qqaq=dirpe0xb9q8qiLsFr0=vr0=vr0dc8meaabaqaciaacaGaaeqabaqabeGadaaakeaacqqGGaaicqWGybawdaqadaqaaiabdsha0bGaayjkaiaawMcaaiabg2da9iabdggaHnaadmaabaGaemiDaqNaeyOeI0YaaSaaaeaacqWGIbGyaeaaiiGacqWFYoGycqWGWbaCaaGagiiBaWMaeiOBa42aaeWaaeaadaWcaaqaaiabigdaXiabgUcaRiabdseaenaaDaaaleaacqaIWaamaeaacqWGWbaCaaaakeaacqaIXaqmcqGHRaWkcqWGebardaqhaaWcbaGaeGimaadabaGaemiCaahaaOGaemyzau2aaWbaaSqabeaacqGHsislcqWGWbaCcqWFYoGycqWG0baDaaaaaaGccaGLOaGaayzkaaaacaGLBbGaayzxaaaaaa@52E3@,

x(t)=e−X(t)[x0+∫0teX(t−τ)S(τ)dτ]     (7)
 MathType@MTEF@5@5@+=feaafiart1ev1aaatCvAUfKttLearuWrP9MDH5MBPbIqV92AaeXatLxBI9gBaebbnrfifHhDYfgasaacH8akY=wiFfYdH8Gipec8Eeeu0xXdbba9frFj0=OqFfea0dXdd9vqai=hGuQ8kuc9pgc9s8qqaq=dirpe0xb9q8qiLsFr0=vr0=vr0dc8meaabaqaciaacaGaaeqabaqabeGadaaakeaacqWG4baEdaqadaqaaiabdsha0bGaayjkaiaawMcaaiabg2da9iabdwgaLnaaCaaaleqabaGaeyOeI0IaemiwaG1aaeWaaeaacqWG0baDaiaawIcacaGLPaaaaaGcdaWadaqaaiabdIha4naaBaaaleaacqaIWaamaeqaaOGaey4kaSYaa8qmaeaacqWGLbqzdaahaaWcbeqaaiabdIfaynaabmaabaGaemiDaqNaeyOeI0ccciGae8hXdqhacaGLOaGaayzkaaaaaOGaem4uam1aaeWaaeaacqWFepaDaiaawIcacaGLPaaacqWGKbazcqWFepaDaSqaaiabicdaWaqaaiabdsha0bqdcqGHRiI8aaGccaGLBbGaayzxaaGaaCzcaiaaxMaadaqadaqaaiabiEda3aGaayjkaiaawMcaaaaa@569A@

**Figure 3:**
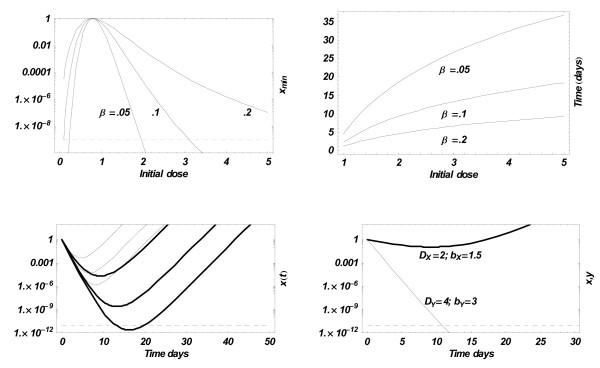
**Pharmaco-dynamics of the simple 'growth-treatment' model**. The upper-left panel shows maximal parasite reduction (8) as a function of (relative) initial dose *D*_0 _for fixed clearing efficiency *b *= 3 and several decay rates *β*. The upper right panel shows the corresponding time to attain 'maximal clearing' (lowest parasite density). The lower left panels plot exact solutions *x*(*t*) for fixed *b *= 3, three choices *D*_0 _= 2,3,4, and two decay rates *β *= .2 (thin curves) and *β *= .1 (thick curves). Only one of them (*β *= .1; *D*_0 _= 4) leads to complete clearing by day 12. The lower right panel compares treatment outcomes of a sensitive strain (thin) and resistant strain (thick) at the decay rate *β *= .05, with clearing rates *b*_*X *_= .5*b*_*Y*_; *D*_*X *_= .5*D*_*Y*_.

This form allows one to estimate the drug efficacy (maximal burden reduction) and the time required to reach it. Assuming zero inoculation (S = 0) in (2) we get

tmin⁡=1βln[(b-1)1/pD0];Xmin⁡=aβp{ln⁡[bb(b−1)b−1]+pln⁡D0−bln⁡(1+D0p)}     (8)
 MathType@MTEF@5@5@+=feaafiart1ev1aaatCvAUfKttLearuWrP9MDH5MBPbIqV92AaeXatLxBI9gBaebbnrfifHhDYfgasaacH8akY=wiFfYdH8Gipec8Eeeu0xXdbba9frFj0=OqFfea0dXdd9vqai=hGuQ8kuc9pgc9s8qqaq=dirpe0xb9q8qiLsFr0=vr0=vr0dc8meaabaqaciaacaGaaeqabaqabeGadaaakeaafaqaaeGabaaabaGaemiDaq3aaSbaaSqaaiGbc2gaTjabcMgaPjabc6gaUbqabaGccqGH9aqpdaWcaaqaaiabigdaXaqaaGGaciab=j7aIbaaieaacqGFSbaBcqGFUbGBdaWadaqaamaabmaabaacbiGae0NyaiMae4xla0Iae4xmaedacaGLOaGaayzkaaWaaWbaaSqabeaacqaIXaqmcqGGVaWlcqWGWbaCaaGccqWGebardaWgaaWcbaGaeGimaadabeaaaOGaay5waiaaw2faaiabcUda7aqaaiabdIfaynaaBaaaleaacyGGTbqBcqGGPbqAcqGGUbGBaeqaaOGaeyypa0ZaaSaaaeaacqWGHbqyaeaacqWFYoGycqWGWbaCaaWaaiWabeaacyGGSbaBcqGGUbGBdaWadaqaamaalaaabaGaemOyai2aaWbaaSqabeaacqWGIbGyaaaakeaadaqadaqaaiabdkgaIjabgkHiTiabigdaXaGaayjkaiaawMcaamaaCaaaleqabaGaemOyaiMaeyOeI0IaeGymaedaaaaaaOGaay5waiaaw2faaiabgUcaRiabdchaWjGbcYgaSjabc6gaUjabdseaenaaBaaaleaacqaIWaamaeqaaOGaeyOeI0IaemOyaiMagiiBaWMaeiOBa42aaeWaaeaacqaIXaqmcqGHRaWkcqWGebardaqhaaWcbaGaeGimaadabaGaemiCaahaaaGccaGLOaGaayzkaaaacaGL7bGaayzFaaaaaiaaxMaacaWLjaWaaeWaaeaacqaI4aaoaiaawIcacaGLPaaaaaa@7A0F@

in terms of its relative clearing *b *> 1, Hill exponent *p*, drug decay-rate *β*, and (relative) initial dose *D*_0_. The (dimensionless) clearing level for malaria is set at *x*_*c *_= 10^-11 ^(relative to the pyrogenic *x*_*p *_= 1), i.e. less than one parasite in the entire 4.5-liter blood volume of an adult. Figure 3 shows (upper left) the maximal parasite removal as a function of the relative initial dose *D*_0 _for three hypothetical drugs with decay rates *β *= 0.2/day, 0.1/day, and 0.05/day; (upper right) the corresponding clearing time *t*_min_; (lower left) the resulting solutions *x*(*t*) for several initial doses *D*_0_. The dashed line at the bottom is the hypothetical (relative) clearing level for malaria parasites. In particular, the decay rate 0.05/day (close to mefloquine) would require an initial dose *d*_0 _= 2*d*_50 _to kill the parasites by day 18. The lower right plot compares the effect of a drug with *β *= 0.05/day on two hypothetical strains: 'sensitive' (thin line) with clearing rate *b*_*Y *_= 3 and relative initial dose *D*_*Y *_= 4, and 'resistant' (thick line) with *b*_*X *_= 1.5, *D*_*X *_= 2. So both parameters (parasite clearing rates and sensitivity thresholds) differ by factor 2. Note that the different relative D's do not imply different actual doses, as both strains inhabit the same 'treated host', but rather refer to different sensitivity thresholds *d*_50 _for *x *and *y*.

This method also works in the case of zero parasite growth (*a *= 0), when equation (2) is solved for initial value *x*_0_. The corresponding solution *x*(*t*) (7) would then stabilize at its minimal value, xmin⁡/x0=(1+D0p)−b/βp
 MathType@MTEF@5@5@+=feaafiart1ev1aaatCvAUfKttLearuWrP9MDH5MBPbIqV92AaeXatLxBI9gBaebbnrfifHhDYfgasaacH8akY=wiFfYdH8Gipec8Eeeu0xXdbba9frFj0=OqFfea0dXdd9vqai=hGuQ8kuc9pgc9s8qqaq=dirpe0xb9q8qiLsFr0=vr0=vr0dc8meaabaqaciaacaGaaeqabaqabeGadaaakeaacqWG4baEdaWgaaWcbaGagiyBa0MaeiyAaKMaeiOBa4gabeaakiabc+caViabdIha4naaBaaaleaacqaIWaamaeqaaOGaeyypa0ZaaeWaaeaacqaIXaqmcqGHRaWkcqWGebardaqhaaWcbaGaeGimaadabaGaemiCaahaaaGccaGLOaGaayzkaaWaaWbaaSqabeaacqGHsislcqWGIbGycqGGVaWliiGacqWFYoGycqWGWbaCaaaaaa@445E@. So the 'clearing dose' to bring parasitaemia down from *x*_0 _below *x*_*c *_is estimated by

D0≥[(x0/xc)βp/b−1]1/p     (9)
 MathType@MTEF@5@5@+=feaafiart1ev1aaatCvAUfKttLearuWrP9MDH5MBPbIqV92AaeXatLxBI9gBaebbnrfifHhDYfgasaacH8akY=wiFfYdH8Gipec8Eeeu0xXdbba9frFj0=OqFfea0dXdd9vqai=hGuQ8kuc9pgc9s8qqaq=dirpe0xb9q8qiLsFr0=vr0=vr0dc8meaabaqaciaacaGaaeqabaqabeGadaaakeaacqWGebardaWgaaWcbaGaeGimaadabeaakiabgwMiZoaadmaabaWaaeWaaeaacqWG4baEdaWgaaWcbaGaeGimaadabeaakiabc+caViabdIha4naaBaaaleaacqWGJbWyaeqaaaGccaGLOaGaayzkaaWaaWbaaSqabeaaiiGacqWFYoGycqWGWbaCcqGGVaWlcqWGIbGyaaGccqGHsislcqaIXaqmaiaawUfacaGLDbaadaahaaWcbeqaaiabigdaXiabc+caViabdchaWbaakiaaxMaacaWLjaWaaeWaaeaacqaI5aqoaiaawIcacaGLPaaaaaa@493E@

While immune regulation complicates the dynamics of drug treatment beyond the simple model (2), it still affords some clues for possible treatment strategies. Indeed, sustained immune levels (I*; J* > 0) in the 'mixed' system (3) or a simpler 'single-strain' version would effectively lower the parasite growth rate from its natural value *a *to *a*' = *a*(1 - *c*_*n*_I* - *c*_*X*_J*). This would automatically raise the clearing drug-efficiency for such an 'immune-competent' host from its 'natural' value *b *to *b*' = *B*/*a*', hence to higher clearing in a shorter time, as illustrated in Figure 3 (lower left panel). This result supports earlier findings [9,10] on the positive impact of acquired immunity on antimalarial therapies.

**Figure 4:**
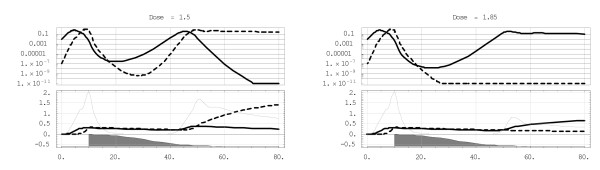
**Single treatment**. The effect of single treatment (shaded region at the bottom) on the *x*-*y *competing pair of Figure 2(a) with different initial dose *D*_*Y *_= *D*_0 _= 1.5 vs. 1.85, over an 80-day time span. The upper plots show (as above) the rescaled *x*,*y *densities (*x*-solid, *y*-dashed), the lower plots the corresponding immune effectors (*J*,*K *– solid/dashed, *I *– thin line).

Unlike this 'simple growth' (2), however, dynamic immune regulation (with or without treatment) renders system (3) unsolvable, so it is analysed using numeric simulations. The analysis of treatment for mixed phenotypes starts with a single drug dose applied at the peak parasitaemia, close to the pyrogenic threshold, and considers two phenotypes with the parameters of Figure 4(a), where *y *dominates and, in the absence of inoculation, drives *x *to extinction. As above we assume different 'drug clearing rates' and sensitivity thresholds for *x*,*y*: *b*_*X *_= 1.5;*b*_*Y *_= 3; *d*_*X *_= 2*d*_*Y *_(so the *x*- strain exhibits substantially higher resistance than *y*). The drug is introduced by day 10, when the 'fast' *y *reaches its peak parasitaemia. The outcomes are shown in Figure 4 for two different values of the relative initial dose *D*_*Y *_= *d*_0_/*d*_*Y*_. For *D*_*Y *_= 1.5 (left plot) both x and *y *are brought to their minima by day 20 and 28 respectively, with *x *taking a temporal lead through day 45. Then the competitive dominance of *y *is restored, and, as in Figure 4(a), leads to the demise of *x *by day 70. Increasing the dose to *D*_*Y *_= 1.85 is sufficient to eliminate *y*, making *x *the sole survivor, by day 27. Note that *D*_*Y *_= 1.5 or 1.85 exceeds the predicted value *D*_0 _= 1.37, if one applies a 'neutral-growth' model (2) initiated at the pyrogenic level *x*_0 _= 1. This example demonstrates important differences between the 'balanced states' of nonlinear interacting parasite-immune-drug systems and the simple linear-growth system (2) often used in pharmacokinetic estimates of dosage.

Thus the above has shown how a high single-treatment dose can lead to the dominance of an otherwise less-fit drug-resistant/tolerant phenotype, in the absence of inoculation. Clearly, the introduction of a *y*-inoculum following such treatment would restore '*y*-domination,' as predicted in Figure 4(a). Successful malaria prophylaxis or treatment in endemically-exposed hosts typically requires repeated doses.

### Repeated periodic treatment

**Figure 5:**
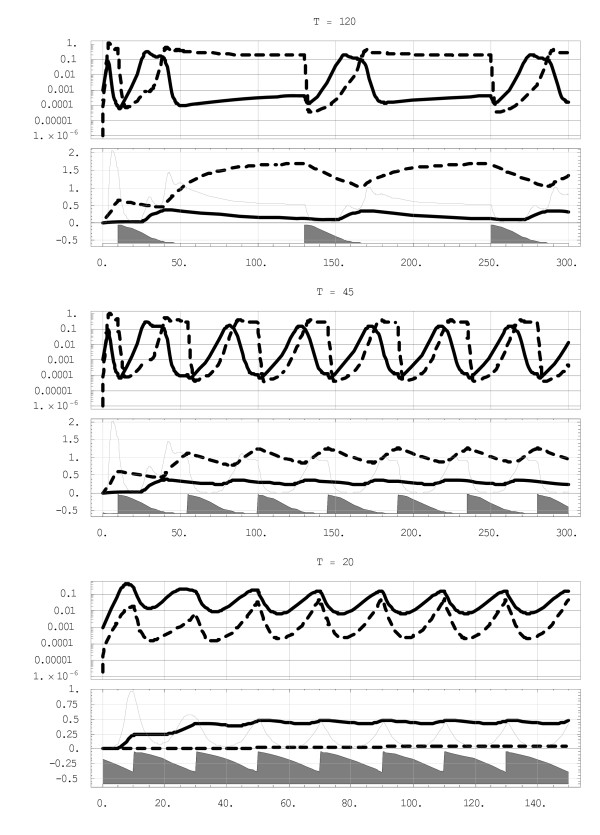
**Effect of periodic treatment on inter-strain competition**. The outcomes vary from long periods of 'y-domination' (top), to a coexistence pattern (middle) to persistent 'x-domination' (bottom). The upper plot has relatively short windows of '*x*-domination', the lower one has persistent 'dominant *x*' by 1 or 2 orders of magnitude over *y*. The treatment cover is shown as gray areas; curve marking and axes are the same as Figure 4.

Now the effect of multiple, periodically-spaced drug doses at time intervals *T *is explored. The drug concentration and its 'clearing function' become periodic as well, namely,

D(t)=D0e−βMod(t,T)1−e−βT     (10)
 MathType@MTEF@5@5@+=feaafiart1ev1aaatCvAUfKttLearuWrP9MDH5MBPbIqV92AaeXatLxBI9gBaebbnrfifHhDYfgasaacH8akY=wiFfYdH8Gipec8Eeeu0xXdbba9frFj0=OqFfea0dXdd9vqai=hGuQ8kuc9pgc9s8qqaq=dirpe0xb9q8qiLsFr0=vr0=vr0dc8meaabaqaciaacaGaaeqabaqabeGadaaakeaacqWGebardaqadaqaaiabdsha0bGaayjkaiaawMcaaiabg2da9iabdseaenaaBaaaleaacqaIWaamaeqaaOWaaSaaaeaacqWGLbqzdaahaaWcbeqaaiabgkHiTGGaciab=j7aIjabb2eanjabb+gaVjabbsgaKnaabmaabaGaemiDaqNaeiilaWIaemivaqfacaGLOaGaayzkaaaaaaGcbaGaeGymaeJaeyOeI0Iaemyzau2aaWbaaSqabeaacqGHsislcqWFYoGycqWGubavaaaaaOGaaCzcaiaaxMaadaqadaqaaiabigdaXiabicdaWaGaayjkaiaawMcaaaaa@4CCD@

where Mod(*t*,*T*) designates a periodic linear function *t *on interval [0,*T*]. Turning to the clearing function *φ*(*D*(*t*)), observe that a high Hill exponent of *φ*(*z*) allows *φ*(*D*(*t*)) to be approximated by a periodic step-function, taking value 1 on interval [0, *T*_*M*_] and 0 on the complimentary range [*T*_*M*_,*T*], with *T*_*M *_≈ ln *D*_0_/*β*. Hence as the first-order approximation the periodic *φ*(*D*(*t*)) can be replaced by its mean value,

φ¯=1T∫0Tφ(D0e−βt)dt≈ln⁡D0βT     (11)
MathType@MTEF@5@5@+=feaafiart1ev1aaatCvAUfKttLearuWrP9MDH5MBPbIqV92AaeXatLxBI9gBaebbnrfifHhDYfgasaacH8akY=wiFfYdH8Gipec8Eeeu0xXdbba9frFj0=OqFfea0dXdd9vqai=hGuQ8kuc9pgc9s8qqaq=dirpe0xb9q8qiLsFr0=vr0=vr0dc8meaabaqaciaacaGaaeqabaqabeGadaaakeaaiiGaliqb=z8aMzaaraGae8xpa0ZaaSaaaeaaiiaacqGFXaqmaeaaieGacqqFubavaaWaa8qCaeaacqWFgpGzaWqaaGqaaiab8bdaWaqaaiab9rfaubGdcqGHRiI8aSWaaeWaaeaacqWGebardaWgaaadbaGaeGimaadabeaaliabdwgaLnaaCaaameqabaGaeyOeI0Iae8NSdiMaemiDaqhaaaWccaGLOaGaayzkaaGaemizaqMaemiDaqNaeyisIS7aaSaaaeaacyGGSbaBcqGGUbGBcqWGebardaWgaaadbaGaeGimaadabeaaaSqaaiab=j7aIjabdsfaubaacaWLjaGaaCzcamaabmaabaGaeGymaeJaeGymaedacaGLOaGaayzkaaaaaa@5244@

where *D*_0 _is either *D*_*X *_or *D*_*Y*_. The long-term effect of such treatment is to effectively lower the parasites' growth-rates to *a*_*Y *_- *b*_*Y*_φ¯
 MathType@MTEF@5@5@+=feaafiart1ev1aaatCvAUfeBSjuyZL2yd9gzLbvyNv2CaerbwvMCKfMBHbqedmvETj2BSbqee0evGueE0jxyaibaieIgFLIOYR2NHOxjYhrPYhrPYpI8F4rqqrFfpeea0xe9Lq=Jc9vqaqpepm0xbbG8FasPYRqj0=yi0lXdbba9pGe9qqFf0dXdHuk9fr=xfr=xfrpiWZqaaeaabiGaaiaacaqabeaabeqacmaaaOqaaGGaciqb=z8aMzaaraaaaa@3755@ and *a*_*X *_- *b*_*X*_φ¯
 MathType@MTEF@5@5@+=feaafiart1ev1aaatCvAUfeBSjuyZL2yd9gzLbvyNv2CaerbwvMCKfMBHbqedmvETj2BSbqee0evGueE0jxyaibaieIgFLIOYR2NHOxjYhrPYhrPYpI8F4rqqrFfpeea0xe9Lq=Jc9vqaqpepm0xbbG8FasPYRqj0=yi0lXdbba9pGe9qqFf0dXdHuk9fr=xfr=xfrpiWZqaaeaabiGaaiaacaqabeaabeqacmaaaOqaaGGaciqb=z8aMzaaraaaaa@3755@. That in turn can modify (reduce) the basic relative growth-rate parameter α=aY−bYφ¯aX−bXφ¯
 MathType@MTEF@5@5@+=feaafiart1ev1aaatCvAUfKttLearuWrP9MDH5MBPbIqV92AaeXatLxBI9gBaebbnrfifHhDYfgasaacH8akY=wiFfYdH8Gipec8Eeeu0xXdbba9frFj0=OqFfea0dXdd9vqai=hGuQ8kuc9pgc9s8qqaq=dirpe0xb9q8qiLsFr0=vr0=vr0dc8meaabaqaciaacaGaaeqabaqabeGadaaakeaaiiGacqWFXoqyiiaacqGF9aqpdaWcaaqaaiabdggaHnaaBaaaleaacqWGzbqwaeqaaOGaeyOeI0IaemOyai2aaSbaaSqaaiabdMfazbqabaGccuWFgpGzgaqeaaqaaiabdggaHnaaBaaaleaacqWGybawaeqaaOGaeyOeI0IaemOyai2aaSbaaSqaaiabdIfaybqabaGccuWFgpGzgaqeaaaaaaa@3FCC@ used in the qualitative analysis of equilibria (see Figure 5). Based on the comparison with the stationary (mean) case, it is to be expected that *α *<*α*_1 _should change y-domination to a (periodically-modulated) coexistence pattern, while *α *<*α*_2 _would bring about 'x- domination'.

Therefore approximate formula (11) is applied to get crude estimates of the treatment frequency for the establishment of the resistant strain. Assuming for simplicity *b*_*X *_= 0 (fully-resistant strain), the equation is obtained for two 'critical' treatment frequencies/periods, *T*_*C *_(for coexistence) and *T*_*R *_(for 'x-domination'),

TC=bln⁡D0β(α−α1)>TR=bln⁡D0β(α−α2);     (12)
 MathType@MTEF@5@5@+=feaafiart1ev1aaatCvAUfKttLearuWrP9MDH5MBPbIqV92AaeXatLxBI9gBaebbnrfifHhDYfgasaacH8akY=wiFfYdH8Gipec8Eeeu0xXdbba9frFj0=OqFfea0dXdd9vqai=hGuQ8kuc9pgc9s8qqaq=dirpe0xb9q8qiLsFr0=vr0=vr0dc8meaabaqaciaacaGaaeqabaqabeGadaaakeaacqWGubavdaWgaaWcbaGaem4qameabeaakiabg2da9maalaaabaGaemOyaiMagiiBaWMaeiOBa4Maemiraq0aaSbaaSqaaiabicdaWaqabaaakeaaiiGacqWFYoGydaqadaqaaiab=f7aHjabgkHiTiab=f7aHnaaBaaaleaacqaIXaqmaeqaaaGccaGLOaGaayzkaaaaaiabg6da+iabdsfaunaaBaaaleaacqWGsbGuaeqaaOGaeyypa0ZaaSaaaeaacqWGIbGycyGGSbaBcqGGUbGBcqWGebardaWgaaWcbaGaeGimaadabeaaaOqaaiab=j7aInaabmaabaGae8xSdeMaeyOeI0Iae8xSde2aaSbaaSqaaiabikdaYaqabaaakiaawIcacaGLPaaaaaGaei4oaSJaaCzcaiaaxMaadaqadaqaaiabigdaXiabikdaYaGaayjkaiaawMcaaaaa@580D@

in terms of pharmacokinetic parameters *β *and b=BaX
 MathType@MTEF@5@5@+=feaafiart1ev1aaatCvAUfKttLearuWrP9MDH5MBPbIqV92AaeXatLxBI9gBaebbnrfifHhDYfgasaacH8akY=wiFfYdH8Gipec8Eeeu0xXdbba9frFj0=OqFfea0dXdd9vqai=hGuQ8kuc9pgc9s8qqaq=dirpe0xb9q8qiLsFr0=vr0=vr0dc8meaabaqaciaacaGaaeqabaqabeGadaaakeaacqWGIbGycqGH9aqpdaWcaaqaaiabdkeacbqaaiabdggaHnaaBaaaleaacqWGybawaeqaaaaaaaa@32CC@, initial dose *D*_0_, and relative growth rate α=aYaX
 MathType@MTEF@5@5@+=feaafiart1ev1aaatCvAUfKttLearuWrP9MDH5MBPbIqV92AaeXatLxBI9gBaebbnrfifHhDYfgasaacH8akY=wiFfYdH8Gipec8Eeeu0xXdbba9frFj0=OqFfea0dXdd9vqai=hGuQ8kuc9pgc9s8qqaq=dirpe0xb9q8qiLsFr0=vr0=vr0dc8meaabaqaciaacaGaaeqabaqabeGadaaakeaaiiGacqWFXoqycqGH9aqpdaWcaaqaaiabdggaHnaaBaaaleaacqWGzbqwaeqaaaGcbaGaemyyae2aaSbaaSqaaiabdIfaybqabaaaaaaa@34D4@. As in section 1 the sensitive strain *y *is assumed competitively superior in the absence of drug, i.e. *α *> *α*_1_, and we expect the following long-term outcomes:

(i) treatment frequency *T *> *T*_*C *_will maintain y-domination;

(ii) *T*_*C *_> *T *> *T*_*R *_will bring about coexistence, with alternating *x*,*y *phases;

(iii) *T *<*T*_*R *_will lead to dominance by the resistant *x*-strain.

To test these predictions the y-dominant case of Figure 2(a) is subjected to random inoculation with EIR = 0.3/day, distributed among 2 strains in proportions *p*_*X *_= .6; *p*_*Y *_= .4 (60% of inoculations are *x*-type, 40% are *y*). Periodic treatment (10) is then applied to such a host, and a range of values of period *T *sampled, using the pharmacokinetic parameters of the two strains as in the previous section, *b*_*X *_= 1.5;*b*_*Y *_= 3; *d*_*x *_= 2*d*_*Y*_.

Figure 5 shows the resulting outcomes, ranging from *y*-dominance at period T = 120 days, to alternating *x *or *y *dominance (T = 45 days; 'periodic coexistence'), to x-dominance at T = 20. The inoculations, though barely visible in plots, still play a role here. Indeed, without them the process would terminate (at T = 120) after the first two cycles. Namely, the first cycle would drop 'treated y' to near-extinction, after which it would rebound, at a lower level, and drive x to extinction (Figure 5, left), but a second treatment would *y *altogether. These numeric results confirm the earlier qualitative predictions on the 'dominance – coexistence' transition, but the 'critical' periods *T*_*C*_;*T*_*R *_differ from the simple estimates (12).

**Figure 6:**
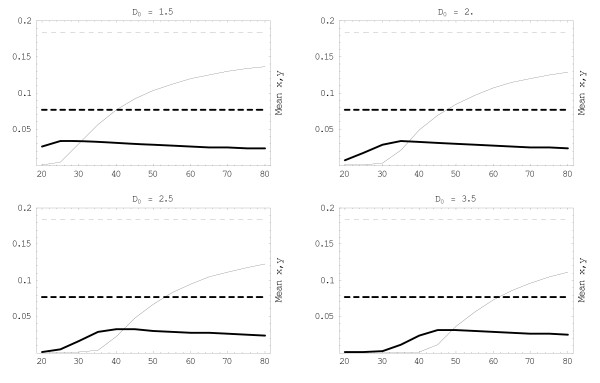
**Time average densities**. Periodically treated mean values x¯
 MathType@MTEF@5@5@+=feaafiart1ev1aaatCvAUfKttLearuWrP9MDH5MBPbIqV92AaeXatLxBI9gBaebbnrfifHhDYfgasaacH8akY=wiFfYdH8Gipec8Eeeu0xXdbba9frFj0=OqFfea0dXdd9vqai=hGuQ8kuc9pgc9s8qqaq=dirpe0xb9q8qiLsFr0=vr0=vr0dc8meaabaqaciaacaGaaeqabaqabeGadaaakeaacuWG4baEgaqeaaaa@2E3D@, y¯
 MathType@MTEF@5@5@+=feaafiart1ev1aaatCvAUfKttLearuWrP9MDH5MBPbIqV92AaeXatLxBI9gBaebbnrfifHhDYfgasaacH8akY=wiFfYdH8Gipec8Eeeu0xXdbba9frFj0=OqFfea0dXdd9vqai=hGuQ8kuc9pgc9s8qqaq=dirpe0xb9q8qiLsFr0=vr0=vr0dc8meaabaqaciaacaGaaeqabaqabeGadaaakeaacuWG5bqEgaqeaaaa@2E3F@ (thick/thin) as functions of period 35 <*T *< 120 for increased initial dose 1.5 <*D*_*Y *_< 3.5. Increased *D*_*Y *_and frequency 1/T facilitate the onset of drug-resistance (dominant x¯
 MathType@MTEF@5@5@+=feaafiart1ev1aaatCvAUfKttLearuWrP9MDH5MBPbIqV92AaeXatLxBI9gBaebbnrfifHhDYfgasaacH8akY=wiFfYdH8Gipec8Eeeu0xXdbba9frFj0=OqFfea0dXdd9vqai=hGuQ8kuc9pgc9s8qqaq=dirpe0xb9q8qiLsFr0=vr0=vr0dc8meaabaqaciaacaGaaeqabaqabeGadaaakeaacuWG4baEgaqeaaaa@2E3D@), e.g. the transition period *T*_*R *_= 30 for *D*_*Y *_= 1.5, is shifted towards *T*_*R *_= 49 for *D*_*Y *_= 3.5. Dashed lines indicate untreated equilibria for *y *(thin) and x (thick). The parameter *α *= 2.06 > *α*_1 _assures '*y*-domination' for the untreated case.

The effect of treatment on mean parasite densities is demonstrated by taking a 'sensitive + fully resistant' pair (with a different set of parameters) and examining the period-average values of parasitaemia, x¯(T)=1T∫nT(n+1)Tx(t)dt
 MathType@MTEF@5@5@+=feaafiart1ev1aaatCvAUfKttLearuWrP9MDH5MBPbIqV92AaeXatLxBI9gBaebbnrfifHhDYfgasaacH8akY=wiFfYdH8Gipec8Eeeu0xXdbba9frFj0=OqFfea0dXdd9vqai=hGuQ8kuc9pgc9s8qqaq=dirpe0xb9q8qiLsFr0=vr0=vr0dc8meaabaqaciaacaGaaeqabaqabeGadaaakeaacuWG4baEgaqeamaabmaabaGaemivaqfacaGLOaGaayzkaaGaeyypa0ZaaSaaaeaacqaIXaqmaeaacqWGubavaaWaa8qmaeaacqWG4baEdaqadaqaaiabdsha0bGaayjkaiaawMcaaiabdsgaKjabdsha0bWcbaGaemOBa4MaemivaqfabaWaaeWaaeaacqWGUbGBcqGHRaWkcqaIXaqmaiaawIcacaGLPaaacqWGubava0Gaey4kIipaaaa@4613@, y¯(T)=1T∫nT(n+1)Ty(t)dt
 MathType@MTEF@5@5@+=feaafiart1ev1aaatCvAUfKttLearuWrP9MDH5MBPbIqV92AaeXatLxBI9gBaebbnrfifHhDYfgasaacH8akY=wiFfYdH8Gipec8Eeeu0xXdbba9frFj0=OqFfea0dXdd9vqai=hGuQ8kuc9pgc9s8qqaq=dirpe0xb9q8qiLsFr0=vr0=vr0dc8meaabaqaciaacaGaaeqabaqabeGadaaakeaacuWG5bqEgaqeamaabmaabaGaemivaqfacaGLOaGaayzkaaGaeyypa0ZaaSaaaeaacqaIXaqmaeaacqWGubavaaWaa8qmaeaacqWG5bqEdaqadaqaaiabdsha0bGaayjkaiaawMcaaiabdsgaKjabdsha0bWcbaGaemOBa4MaemivaqfabaWaaeWaaeaacqWGUbGBcqGHRaWkcqaIXaqmaiaawIcacaGLPaaacqWGubava0Gaey4kIipaaaa@4617@ and their dependence on T. The relative initial dose 2.5 <*D*_*Y *_< 6 is also varied. In each plot in Figure 6, observe that high treatment-frequency (short *T*) drives *y *to extinction and leaves the dominant *x *at (or close to) its equilibrium value. But an increased initial dose *D*_*Y *_= *D*_0 _extends the frequency range for *x*-domination, from *T *≈ 42 days at *D*_0 _= 3.5, to *T *= 60 at *D*_0 _= 6. It can be shown that the competition model (3) with periodic coefficients (due e.g. to 'periodic treatment') has a stable 'periodic equilibrium' – a counterpart of the stable 'stationary' equilibrium.

The above shows that persistent treatment creates a new 'effective environment' that could significantly alter the time-averaged distribution of phenotype densities within a host. Provided that such 'over-treated' hosts make up a sizable fraction of the population and that transmission to mosquitoes is in some way proportional to parasite densities, this could further promote the spread of drug resistance through the community.

### Stationary treatment

The above has demonstrated some critical effects of periodic drug treatment and superinfection on immune-mediated competition. Many applications require a more 'exact' account of the relationships among all these factors, however. Specifically, this section examines the effects of (i) drug efficiencies *b*_*X *_≪ *b*_*Y*_, (ii) treatment intensity, as measured by the (period average) factor φ¯
 MathType@MTEF@5@5@+=feaafiart1ev1aaatCvAUfeBSjuyZL2yd9gzLbvyNv2CaerbwvMCKfMBHbqedmvETj2BSbqee0evGueE0jxyaibaieIgFLIOYR2NHOxjYhrPYhrPYpI8F4rqqrFfpeea0xe9Lq=Jc9vqaqpepm0xbbG8FasPYRqj0=yi0lXdbba9pGe9qqFf0dXdHuk9fr=xfr=xfrpiWZqaaeaabiGaaiaacaqabeaabeqacmaaaOqaaGGaciqb=z8aMzaaraaaaa@3755@ (11), and (iii) superinfection sources {*S*_*X*_, *S*_*Y*_}, through analysis of an (approximate) *stationary system *obtained by averaging the sources and the treatment regimen. The resulting stationary model allows all three factors to be incorporated in a simple and efficient way.

The 'averaging' proceeds by replacing the stochastic inoculation source by its steady (mean) value (*S*_*X*_, *S*_*Y*_) = (*p*_*X*_, *p*_*Y*_) S¯
 MathType@MTEF@5@5@+=feaafiart1ev1aaatCvAUfKttLearuWrP9MDH5MBPbIqV92AaeXatLxBI9gBaebbnrfifHhDYfgasaacH8akY=wiFfYdH8Gipec8Eeeu0xXdbba9frFj0=OqFfea0dXdd9vqai=hGuQ8kuc9pgc9s8qqaq=dirpe0xb9q8qiLsFr0=vr0=vr0dc8meaabaqaciaacaGaaeqabaqabeGadaaakeaacuWGtbWugaqeaaaa@2DF3@, where S¯
 MathType@MTEF@5@5@+=feaafiart1ev1aaatCvAUfKttLearuWrP9MDH5MBPbIqV92AaeXatLxBI9gBaebbnrfifHhDYfgasaacH8akY=wiFfYdH8Gipec8Eeeu0xXdbba9frFj0=OqFfea0dXdd9vqai=hGuQ8kuc9pgc9s8qqaq=dirpe0xb9q8qiLsFr0=vr0=vr0dc8meaabaqaciaacaGaaeqabaqabeGadaaakeaacuWGtbWugaqeaaaa@2DF3@ is the product of EIR and 'mean inocula' (merozoite release *s*_0_), and (*p*_*X*_, *p*_*Y*_) – the relative fractions of the two phenotypes. By the same pattern we replace the 'periodic drug function' *φ *[*D*(*t*)] with its mean value (treatment intensity) (11), φ¯
 MathType@MTEF@5@5@+=feaafiart1ev1aaatCvAUfeBSjuyZL2yd9gzLbvyNv2CaerbwvMCKfMBHbqedmvETj2BSbqee0evGueE0jxyaibaieIgFLIOYR2NHOxjYhrPYhrPYpI8F4rqqrFfpeea0xe9Lq=Jc9vqaqpepm0xbbG8FasPYRqj0=yi0lXdbba9pGe9qqFf0dXdHuk9fr=xfr=xfrpiWZqaaeaabiGaaiaacaqabeaabeqacmaaaOqaaGGaciqb=z8aMzaaraaaaa@3755@ = φ¯
 MathType@MTEF@5@5@+=feaafiart1ev1aaatCvAUfeBSjuyZL2yd9gzLbvyNv2CaerbwvMCKfMBHbqedmvETj2BSbqee0evGueE0jxyaibaieIgFLIOYR2NHOxjYhrPYhrPYpI8F4rqqrFfpeea0xe9Lq=Jc9vqaqpepm0xbbG8FasPYRqj0=yi0lXdbba9pGe9qqFf0dXdHuk9fr=xfr=xfrpiWZqaaeaabiGaaiaacaqabeaabeqacmaaaOqaaGGaciqb=z8aMzaaraaaaa@3755@(*T*,*D*_0_); 0 ≤ φ¯
 MathType@MTEF@5@5@+=feaafiart1ev1aaatCvAUfeBSjuyZL2yd9gzLbvyNv2CaerbwvMCKfMBHbqedmvETj2BSbqee0evGueE0jxyaibaieIgFLIOYR2NHOxjYhrPYhrPYpI8F4rqqrFfpeea0xe9Lq=Jc9vqaqpepm0xbbG8FasPYRqj0=yi0lXdbba9pGe9qqFf0dXdHuk9fr=xfr=xfrpiWZqaaeaabiGaaiaacaqabeaabeqacmaaaOqaaGGaciqb=z8aMzaaraaaaa@3755@ ≤ 1, considered as a function of the initial dose *D*_*Y *_= *D*_0 _and treatment period *T*. The resulting 'average' equilibrium system is similar to (4),

x[(φ¯−1bX)+1bX(xm1+yn1)]=S1/bX;y[(φ¯−1bY)+1bY(xm2+yn2)]=S2/bY     (13)
 MathType@MTEF@5@5@+=feaafiart1ev1aaatCvAUfKttLearuWrP9MDH5MBPbIqV92AaeXatLxBI9gBaebbnrfifHhDYfgasaacH8akY=wiFfYdH8Gipec8Eeeu0xXdbba9frFj0=OqFfea0dXdd9vqai=hGuQ8kuc9pgc9s8qqaq=dirpe0xb9q8qiLsFr0=vr0=vr0dc8meaabaqaciaacaGaaeqabaqabeGadaaakqaabeqaaiabdIha4naadmaabaWaaeWaaeaaiiGacuWFgpGzgaqeaiab=jHiTmaalaaabaGaeGymaedabaGaemOyai2aaSbaaSqaaiabdIfaybqabaaaaaGccaGLOaGaayzkaaGaey4kaSYaaSaaaeaacqaIXaqmaeaacqWGIbGydaWgaaWcbaGaemiwaGfabeaaaaGcdaqadaqaamaalaaabaGaemiEaGhabaGaemyBa02aaSbaaSqaaiabigdaXaqabaaaaOGaey4kaSYaaSaaaeaacqWG5bqEaeaacqWGUbGBdaWgaaWcbaGaeGymaedabeaaaaaakiaawIcacaGLPaaaaiaawUfacaGLDbaacqGH9aqpcqWGtbWudaWgaaWcbaGaeGymaedabeaakiabc+caViabdkgaInaaBaaaleaacqWGybawaeqaaOGaei4oaSdabaGaemyEaK3aamWaaeaadaqadaqaaiqb=z8aMzaaraGae8NeI0YaaSaaaeaacqaIXaqmaeaacqWGIbGydaWgaaWcbaGaemywaKfabeaaaaaakiaawIcacaGLPaaacqGHRaWkdaWcaaqaaiabigdaXaqaaiabdkgaInaaBaaaleaacqWGzbqwaeqaaaaakmaabmaabaWaaSaaaeaacqWG4baEaeaacqWGTbqBdaWgaaWcbaGaeGOmaidabeaaaaGccqGHRaWkdaWcaaqaaiabdMha5bqaaiabd6gaUnaaBaaaleaacqaIYaGmaeqaaaaaaOGaayjkaiaawMcaaaGaay5waiaaw2faaiabg2da9iabdofatnaaBaaaleaacqaIYaGmaeqaaOGaei4la8IaemOyai2aaSbaaSqaaiabdMfazbqabaGccaWLjaGaaCzcamaabmaabaGaeGymaeJaeG4mamdacaGLOaGaayzkaaaaaaa@759C@

But somewhat different rescaling and notations are used for the '*x*,*y*'-intercepts (designated here by {*m*_*i*_, *n*_*i*_}), and for the stationary (mean) sources (*S*_1_,*S*_2_) = (*αp*_*X*_, *p*_*Y*_)*S *of relative strength *S *= EIR × s_0_/*a*_*Y*_, where *α *= *a*_*Y*_/*a*_*x *_– the above 'fitness' parameter. All three factors (intensity φ¯
 MathType@MTEF@5@5@+=feaafiart1ev1aaatCvAUfeBSjuyZL2yd9gzLbvyNv2CaerbwvMCKfMBHbqedmvETj2BSbqee0evGueE0jxyaibaieIgFLIOYR2NHOxjYhrPYhrPYpI8F4rqqrFfpeea0xe9Lq=Jc9vqaqpepm0xbbG8FasPYRqj0=yi0lXdbba9pGe9qqFf0dXdHuk9fr=xfr=xfrpiWZqaaeaabiGaaiaacaqabeaabeqacmaaaOqaaGGaciqb=z8aMzaaraaaaa@3755@, clearing rates *b*_*x *_≪ *b*_*Y*_, and sources {*S*_*i*_}) enter (13) explicitly. The resulting 'stationary equilibria' of (13) are expected to approximate the stable periodic (or quasi-periodic/stochastic) equilibria of the original system.

The exact solution of the 4-th order algebraic system (13) is given by (grossly cumbersome) functions: *x *= x* (φ¯
 MathType@MTEF@5@5@+=feaafiart1ev1aaatCvAUfeBSjuyZL2yd9gzLbvyNv2CaerbwvMCKfMBHbqedmvETj2BSbqee0evGueE0jxyaibaieIgFLIOYR2NHOxjYhrPYhrPYpI8F4rqqrFfpeea0xe9Lq=Jc9vqaqpepm0xbbG8FasPYRqj0=yi0lXdbba9pGe9qqFf0dXdHuk9fr=xfr=xfrpiWZqaaeaabiGaaiaacaqabeaabeqacmaaaOqaaGGaciqb=z8aMzaaraaaaa@3755@, *S,b*_*X*_,*b*_*Y*_) and *y *= y* (*φ*,*S,b*_*X*_,*b*_*Y*_). (Note that these can be brought into a more manageable 'analytic form' under simplifying assumptions, e.g. a 'highly sensitive' *y*-strain.) These can easily be manipulated, however (for numeric and graphic purposes), by any symbolic algebra package (here Mathematica 5). Having four independent parameters in x*, y* some of them can be fixed (e.g. clearing rates *b*_*Y*_;*b*_*X *_– for the drug-sensitive/resistant phenotypes), to examine the effect of the treatment intensity 0 <φ¯
 MathType@MTEF@5@5@+=feaafiart1ev1aaatCvAUfeBSjuyZL2yd9gzLbvyNv2CaerbwvMCKfMBHbqedmvETj2BSbqee0evGueE0jxyaibaieIgFLIOYR2NHOxjYhrPYhrPYpI8F4rqqrFfpeea0xe9Lq=Jc9vqaqpepm0xbbG8FasPYRqj0=yi0lXdbba9pGe9qqFf0dXdHuk9fr=xfr=xfrpiWZqaaeaabiGaaiaacaqabeaabeqacmaaaOqaaGGaciqb=z8aMzaaraaaaa@3755@ < 1, and/or the source strength *S*.

**Figure 7:**
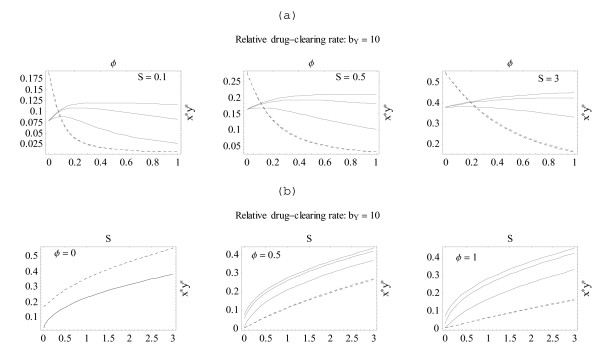
**Equilibria of stationary model**. Three plots of panel (a) show equilibria of system (13) (*x*-solid, *y*-dashed) as functions of relative treatment intensity 0 <*φ *< 1, for selected values of the superinfection source *S*. Panel (b) does the same in terms of the variable 0 <*S *< 3 (source strength) for 3 selected treatment intensities. Three solid curves on each plot (in descending order) correspond to 3 values of clearing rate for resistant strain: *b*_*X *_= 0.25 (highly resistant), 1 (medium); 4 (low).

Figure 7(a) shows equilibria x*,y* as functions of treatment intensity 0 <φ¯
 MathType@MTEF@5@5@+=feaafiart1ev1aaatCvAUfeBSjuyZL2yd9gzLbvyNv2CaerbwvMCKfMBHbqedmvETj2BSbqee0evGueE0jxyaibaieIgFLIOYR2NHOxjYhrPYhrPYpI8F4rqqrFfpeea0xe9Lq=Jc9vqaqpepm0xbbG8FasPYRqj0=yi0lXdbba9pGe9qqFf0dXdHuk9fr=xfr=xfrpiWZqaaeaabiGaaiaacaqabeaabeqacmaaaOqaaGGaciqb=z8aMzaaraaaaa@3755@ < 1, and Figure 7(b) does the same in terms of the source strength 0 <*S *< 3. In the upper plots (a) we fix *b*_*Y *_= 10 (for the drug-sensitive strain), and take three dispersed values of the source strength *S*. The lower plots (b) show a similar exercise for x*,y* as functions of source strength *S *for three treatment intensities. Three solid curves on each panel correspond to three choices of *b*_*X *_: highly drug-resistant *b*_*X *_= .25 (top solid curve), moderate *b*_*X *_= 1 (middle curve), and relatively sensitive *b*_*X *_= 4 (bottom curve). The overall effect of increased intensity φ¯
 MathType@MTEF@5@5@+=feaafiart1ev1aaatCvAUfeBSjuyZL2yd9gzLbvyNv2CaerbwvMCKfMBHbqedmvETj2BSbqee0evGueE0jxyaibaieIgFLIOYR2NHOxjYhrPYhrPYpI8F4rqqrFfpeea0xe9Lq=Jc9vqaqpepm0xbbG8FasPYRqj0=yi0lXdbba9pGe9qqFf0dXdHuk9fr=xfr=xfrpiWZqaaeaabiGaaiaacaqabeaabeqacmaaaOqaaGGaciqb=z8aMzaaraaaaa@3755@ is to bring down y* (by several orders of magnitude), while maintaining near-stable x*. In the upper panel (a), *x *becomes dominant at relatively low treatment levels; this threshold φ¯
 MathType@MTEF@5@5@+=feaafiart1ev1aaatCvAUfeBSjuyZL2yd9gzLbvyNv2CaerbwvMCKfMBHbqedmvETj2BSbqee0evGueE0jxyaibaieIgFLIOYR2NHOxjYhrPYhrPYpI8F4rqqrFfpeea0xe9Lq=Jc9vqaqpepm0xbbG8FasPYRqj0=yi0lXdbba9pGe9qqFf0dXdHuk9fr=xfr=xfrpiWZqaaeaabiGaaiaacaqabeaabeqacmaaaOqaaGGaciqb=z8aMzaaraaaaa@3755@_*c *_increases with source strength (0 <*S *< 3), but remains confined within a narrow range .1 <φ¯
 MathType@MTEF@5@5@+=feaafiart1ev1aaatCvAUfeBSjuyZL2yd9gzLbvyNv2CaerbwvMCKfMBHbqedmvETj2BSbqee0evGueE0jxyaibaieIgFLIOYR2NHOxjYhrPYhrPYpI8F4rqqrFfpeea0xe9Lq=Jc9vqaqpepm0xbbG8FasPYRqj0=yi0lXdbba9pGe9qqFf0dXdHuk9fr=xfr=xfrpiWZqaaeaabiGaaiaacaqabeaabeqacmaaaOqaaGGaciqb=z8aMzaaraaaaa@3755@_*c *_< .2. In the lower panel (b), equilibria increase with the source strength *S *(as expected), and, at *φ *= .5, the *x*-strain becomes fully dominant. Note that in both panels widely divergent drug sensitivities *b*_*X *_of the *x*-strain have only marginal effects on the behaviour of *y*-equilibria (either its φ¯
 MathType@MTEF@5@5@+=feaafiart1ev1aaatCvAUfeBSjuyZL2yd9gzLbvyNv2CaerbwvMCKfMBHbqedmvETj2BSbqee0evGueE0jxyaibaieIgFLIOYR2NHOxjYhrPYhrPYpI8F4rqqrFfpeea0xe9Lq=Jc9vqaqpepm0xbbG8FasPYRqj0=yi0lXdbba9pGe9qqFf0dXdHuk9fr=xfr=xfrpiWZqaaeaabiGaaiaacaqabeaabeqacmaaaOqaaGGaciqb=z8aMzaaraaaaa@3755@ – or *S*-dependence).

This suggests that treatment intensity is more crucial for the onset of resistance than inoculation frequency. The increased source by itself (at all treatment levels) would raise equilibria but diminish their relative difference: x*/y* ≈ 1 at high *S*.

## Discussion

The above has examined how immune-mediated competition between parasites is perturbed by a drug to which the competing parasites are differentially resistant, and how drug dose, drug timing, and inoculations of new parasites affect these interactions. For clarity, the examples focused on the situation in which, in the absence of drug, the drug-sensitive strain is numerically dominant in immune-mediated competition, but the model spans the full range of possibilities. That is, while the outcome of immune-mediated competition is either that one phenotype dominates, or the phenotypes coexist, further possibilities arise when phenotypes of differing drug sensitivity are subjected to treatment and superinfection. Figure 7 illustrates possible outcomes for a range of sensitivities, drug efficacies, treatment intensities, and superinfection frequencies, and shows the potential for comparing model predictions across the range of possibilities that may arise in empirical studies.

Actual frequencies of parasite inoculation vary with the size of the vector mosquito population, and, like the composition of inocula, with the prevalence and characteristics of parasites and immunity in the human population. The baseline results reflect an inoculum that initiates an infection, i.e. after any previous infections have cleared. The parasites in superinfecting inocula are in general greatly outnumbered by those in an ongoing infection, particularly when the ongoing infection is at or near its peak. In the model, parasite inoculation enters either as a random (stochastic) source, or as its stationary (mean) value. The latter allowed exploration of a broad range of source strengths (EIR): overall, increased strength drives both equilibrium densities (or their 'period means') up, but increased intensity and efficacy of treatment eventually tips the outcome from the strain that is more 'fit' in terms of a host immune response to the one that is more 'fit' in terms of a drug.

What is transmitted from an infected human to a mosquito, and onward, poses another complex set of questions, but our results here seem to support the hypothesis that immune-mediated interactions can shape the spread of drug resistance, even if the phenotypic traits are not linked genetically [19,20]. The fitness cost of drug resistance, considered here simply in terms of replication rate, is likely to be multifactorial in a population of hosts heterogeneous with respect to infection histories and immune profiles [21]. It would be interesting, in future work, to explicitly consider the genetics of parasite drug resistance, with respect to both origin and spread, and the common use of anti-malarial drugs to "treat" non-malarial fevers or other symptoms.

Antigenic variation can also play a role in parasite competition. From the standpoint of this model the only potentially relevant effect is a change of immune stimulation/clearing, since parasites' intrinsic replication or drug sensitivities should not be affected. A simple way to accommodate antigenic variation (in lieu of more complicated 'multi-strain/multi-clone' models [22,23]) is to make the specific immune clearing function to change in time *c*(*t*). A drop in *c *can be thought of as resulting from a new variant of 'low cross-reactivity' to prior antibodies, taking over and growing into a dominant strain. As the clone keeps replicating, specific antibodies develop, and function *c*(*t*) gradually 'relaxes' to its normal (relatively high) value. Such a 'random drop + relaxation' form of *c*(*t*) was proposed in [13].

Time dependent *c*(*t*) would make the model non-stationary, the same way as the 'variable inoculation source' or 'non-stationary treatment'. In each case the strategy here was to 'average variable coefficients' over time. Applying the same 'averaging methodology' to variable clearing rates would replace them with somewhat lower 'mean values' {c¯
 MathType@MTEF@5@5@+=feaafiart1ev1aaatCvAUfKttLearuWrP9MDH5MBPbIqV92AaeXatLxBI9gBaebbnrfifHhDYfgasaacH8akY=wiFfYdH8Gipec8Eeeu0xXdbba9frFj0=OqFfea0dXdd9vqai=hGuQ8kuc9pgc9s8qqaq=dirpe0xb9q8qiLsFr0=vr0=vr0dc8meaabaqaciaacaGaaeqabaqabeGadaaakeaacuWGJbWygaqeaaaa@2E13@_*X*_; c¯
 MathType@MTEF@5@5@+=feaafiart1ev1aaatCvAUfKttLearuWrP9MDH5MBPbIqV92AaeXatLxBI9gBaebbnrfifHhDYfgasaacH8akY=wiFfYdH8Gipec8Eeeu0xXdbba9frFj0=OqFfea0dXdd9vqai=hGuQ8kuc9pgc9s8qqaq=dirpe0xb9q8qiLsFr0=vr0=vr0dc8meaabaqaciaacaGaaeqabaqabeGadaaakeaacuWGJbWygaqeaaaa@2E13@_*Y *_}, and the new 'effective' *c *would enter formulae and analyses. Indeed, lower {*e*_*X*_;*e*_*Y*_} (proportional to {c¯
 MathType@MTEF@5@5@+=feaafiart1ev1aaatCvAUfKttLearuWrP9MDH5MBPbIqV92AaeXatLxBI9gBaebbnrfifHhDYfgasaacH8akY=wiFfYdH8Gipec8Eeeu0xXdbba9frFj0=OqFfea0dXdd9vqai=hGuQ8kuc9pgc9s8qqaq=dirpe0xb9q8qiLsFr0=vr0=vr0dc8meaabaqaciaacaGaaeqabaqabeGadaaakeaacuWGJbWygaqeaaaa@2E13@_*X*_;c¯
 MathType@MTEF@5@5@+=feaafiart1ev1aaatCvAUfKttLearuWrP9MDH5MBPbIqV92AaeXatLxBI9gBaebbnrfifHhDYfgasaacH8akY=wiFfYdH8Gipec8Eeeu0xXdbba9frFj0=OqFfea0dXdd9vqai=hGuQ8kuc9pgc9s8qqaq=dirpe0xb9q8qiLsFr0=vr0=vr0dc8meaabaqaciaacaGaaeqabaqabeGadaaakeaacuWGJbWygaqeaaaa@2E13@_*Y*_}) would change (decrease) 'fitness thresholds' {*α*_1_,*α*_2_}, and drive up equilibrium levels of *x*,*y *(as both are cleared less 'efficiently' on average).

Thus the implication of antigenic variation is that degrees of cross-reactivity between parasites may fluctuate during the course of an infection, and its net effect would be to (effectively) lower the immune efficiencies, and change the related equilibria, the fitness ranges of 'domination and coexistence' etc. The major points of interest here have to do with the relationship between the parasite entities, however, which would shift if antigenic variation differs between them in some systematic, biased way, which would then be expressed in terms of relative competitive advantage. Thus most of our results would maintain their qualitative form, but some quantitative changes on the predicted outcomes of treatment would be expected (Figures 6, 7). It might prove interesting, in future work, to explicitly consider the extent to which antigenic variation gives rise to fevers which give rise to drug-taking, for instance.

Apparently no previous work has examined these critical within-host interactions between parasite, immune-response and drug dynamics in a malaria infection, but several models have touched on important aspects of these analyses or on closely-related issues. Davis and Martin [24] compared several simple descriptive models of parasite clearance dynamics during curative drug treatment. Based on the results of pharmacokinetic-pharmacodynamic models, Hoshen et al. [25] argued that well-timed follow-up doses might eliminate even resistant phenotypes, and Simpson et al. [15] that, to prevent resistance, larger doses should be deployed as standard at the first introduction. While these models did not consider host immune responses or parasite replication, Austin et al. [14] noted that "most drugs act best against replicating pathogens in combination with effective immunological responses," joined a pharmacokinetic model to a simple model of pathogen-immune dynamics [26], and derived drug doses and frequencies necessary to reduce the average lifespan of infected RBCs below a critical threshold. Their model did not consider drug- or immune-mediated competition between strains, however.

Gatton et al. [27] modeled the risk of a drug-resistant mutant arising during an infection, in terms of the rate at which a single parasite genotype switches between antigenic variants, the response rate of the corresponding specific antibody, and a simple time-of-treatment model. They found mutants most likely to arise in hosts lacking any such specific antibodies, in hosts treated before antibody response to a switch of variants, and, in recent work [28], in hosts taking drugs with long half-lives or in subcurative doses. Hastings [29] recently developed parasite-population-genetic models that represent competing forces behind shifts in equilibrium gene frequencies in terms of within-host averages and proportions; though these models do not yet encompass considerations of immune responses or other dynamic factors, he emphasized that "intense competition between separate malaria clones co-infecting the same human can generate complex dynamics," and that "the dynamics underlying the evolution of antimalarial resistance may therefore be much more complex than previously realized." This appears irrefutable, hence the model above was developed to address these "complex dynamics."

## Authors' contributions

Mathematical models and analyses were developed jointly by DG and FEM. DG implemented them for computation and ran numeric simulations using Mathematica 5. FEM provided the context, background material and references. Both authors wrote, read and approved the final manuscript.
